# The Royal Infirmary, Dundee

**Published:** 1902-12-20

**Authors:** 


### THE ROYAL INFIRMARY, DUNDEE.

A Correspondent writes: With reference to the paragraph in last week's issue regarding the Dundee Royal Infirmary, we are informed that at the meeting of the Works Committee of the Dundee Town Council, held on October 7th, at which the plans for the additional wards proposed to be erected by Mr. J. K. Caird were disapproved, a protest was lodged against members of the Town Council who were not members of the Works Committee, taking part in the voting. Had the votes of these non-members of committee not been counted the plans would have been approved. The whole matter was reconsidered at a full meeting of the Town Council on October 17th, and on a vote being taken the decision of the Works Committee was reversed by a majority of 16 to 8—the plans being accordingly declared approved. As some doubt existed in the minds of the directors of the Infirmary in regard to the legality of this decision, although apparently favourable to their views, they determined to put the matter beyond dispute by a reference to the Court of Appeal—in this case the Court of Session. An appeal was therefore intimated and on the case being called on October 22nd, Counsel for the Town Council agreed to judgment being entered in favour of the appellants without debate. The plans having thus received the necessary approval, it is expected Mr. Caird will give instructions for building to be begun at an early date.

